# Connection between two historical tuberculosis outbreak sites in Japan, Honshu, by a new *ancestral Mycobacterium tuberculosis* L2 sublineage

**DOI:** 10.1017/S0950268822000048

**Published:** 2022-01-19

**Authors:** Christophe Guyeux, Gaetan Senelle, Guislaine Refrégier, Florence Bretelle-Establet, Emmanuelle Cambau, Christophe Sola

**Affiliations:** 1 DISC Computer Science Department, FEMTO-ST Institute, UMR 6174 CNRS, Univ. Bourgogne Franche-Comté (UBFC), 16 Route de Gray, 25000 Besançon, France; 2 Université Paris-Saclay, Saint-Aubin, France; 3 Université Paris-Saclay, CNRS, AgroParisTech, UMR ESE, 91405, Orsay, France; 4 Université de Paris, SPHERE, UMR7219, CNRS, Paris, France; 5 Université de Paris, IAME, UMR1137, INSERM, Paris, France; 6 AP-HP, GHU Nord, service de mycobactériologie spécialisée et de référence, Laboratoire associé du Centre National de Référence des mycobactéries et résistance des mycobactéries aux antituberculeux (CNR-MyRMA), Paris, France

**Keywords:** Tuberculosis, evolutionary genomics, Japan, Thailand, lineage 2

## Abstract

By gathering 680 publicly available Sequence Read Archives from isolates of *Mycobacterium tuberculosis* complex (MTBC) including 190 belonging to the lineage 2 *Beijing*, and using an in-house bioinformatical pipeline, the *TB-Annotator*, that analyses more than 50 000 characters, we describe herein a new L2 sublineage from 20 isolates found in the Tochigi province, (Japan), that we designate as *asia ancestral 5* (AAnc5). These isolates harbour a number of specific criteria (42 SNPs) and their intra-cluster pairwise distance suggests historical and not epidemiological transmission. These isolates harbour a mutation in *rpoC*, and do not fulfil, any of the *modern Beijing* lineage criteria, nor any of the other *ancestral Beijing* lineages described so far. *Asia ancestral 5* isolates do not possess *mutT2* 58 and *ogt* 12 characteristics of *modern Beijing*, but possess *ancestral Beijing* SNPs characteristics. By looking into the literature, we found a reference isolate ID381, described in Kobe and Osaka belonging to the ‘G3’ group, sharing 36 out of the 42 specific SNPs found in AAnc5. We also assessed the intermediate position of the *asia ancestral 4* (AAnc4) sublineage recently described in Thailand and propose an improved classification of the L2 that now includes AAnc4 and AAnc5. By increasing the recruitment into *TB-Annotator* to around 3000 genomes (including 642 belonging to L2), we confirmed our results and discovered additional historical *ancestral* L2 branches that remain to be investigated in more detail. We also present, in addition, some anthropological and historical data from Chinese and Japan history of tuberculosis, as well as from Korea, that could support our results on L2 evolution. This study shows that the reconstruction of the early history of tuberculosis in Asia is likely to reveal complex patterns since its emergence.

## Introduction

With 9.9 million new cases in 2019, and 500 000 multi-drug resistant cases, tuberculosis (TB) is far from being eradicated [1]. Among 9 acknowledged lineages (L1 to L9) described in *Mycobacterium tuberculosis* complex, the lineage L2 is of great interest [2–6]. Very large outbreaks in L2 were shown to have independently emerged worldwide [7]. Although L2 origin is suspected to be in China and L2 is predominant in east Asia, its exact place and time of emergence are still highly debated [8–10]. L2 clinical isolates have developed specific virulence and drug-resistance features that contribute to their epidemic success [6, 11–13]. From an evolutionary standpoint, L2 has developed a high IS*6110* copy number lifestyle, that could have fostered a hypermutator phenotype that may have increased the virulence or fitness of some of these isolates [14, 15]. The epidemic burst of L2 was first detected during the 90s and was promoted by historical and geopolitical events such as (1) the fall of the former USSR and changes in China; (2) a poorly individualised TB treatment and medical follow-up of prisoners in these countries; (3) the increase in the global trade share of China [16–18]. L2 fostered many research studies to understand multidrug resistance emergence and to improve its genetic characterisation [4, 19, 20]. This was achieved progressively and through the combination of polymorphic markers analysis such as IS*6110*-RFLP^1^, MIRU-VNTR^2^, Regions of Difference (RD), hypervariable VNTR loci, and lastly whole-genome sequencing (WGS) [21–27].

The L2 lineage was split into two main sublineages, L2.1 (Proto-Beijing) and L2.2 (Beijing), [28–31]. L2.1 was described mainly in south-China, particularly in the Guangxi province and could be as ancient as 30 000 years old, having co-evolved with east Asian populations [8]. Among its characteristics, it harbours the RD105 deletion but not RD207 [32]. Rare L2.1 isolates were shown to have become ultra-drug resistant [32]. L2.2, is defined by SIT1^3^ or variants, and is composed of several sublineages, *ancestral* and *modern* ones. L2.2.2 defines the *asia ancestral* 1 sublineage; L2.2.1 gathers all others [33]. RD181 is specifically deleted in all L2.2.1 sublineages [28]. The switch from *ancestral* to *modern* Beijing is associated to the presence of at least one IS*6110* copy in the so-called NTF region and the presence of mutations in replication-repair-recombination (3R) genes, among which the *mutT2* G->C mutation in position 1286766 and the *ogt* C->T mutation at codon 12 position 1477596, relatively to the MTBC H37Rv reference sequence (NC_000962.3) [11, 28, 34]. Until recently *ancestral Beijing* included 3 *asia ancestral* lineages (*asia ancestral 1*, *2* and *3*, AAnc1, AAnc2, AAnc3) [28], until a new *asia ancestral 4* (AAnc4), was discovered in northern Thailand [35]. *Modern* Beijing strains are responsible of most but not of all of the recent MDR-TB outbreaks [4, 15, 36].

The large databases built with WGS data allow to develop a precise and comprehensive knowledge of L2 diversity [4, 26, 31, 37]. Computational genomics now allows to study *in-depth* both the global and the local L2 history [6, 24, 26, 32, 35, 38–42]. The evolutionary history of L2 isolates is still debated as is their precise dating of emergence and their geographical origin [26, 34]. Luo *et al*. suggested that L2 could be as ancient as 30 000 years old [8]. Merker *et al*. estimated the time to the most recent common ancestor (TMRCA) of ~6100 and 5200 years before present (BP) for MIRU-VNTR defined clonal complexes BL7 and CC6 (the most ancient) and ~1500 years BP for CC5 (the most recent) [26]. Liu *et al*. estimated the coalescence between L2.1/Proto-Beijing and L2.2.2/*ancestral* Beijing at 2200 BP, and at 1300 years BP for the split between all *ancestral* Beijing lineages [10]. L2.1/Proto-Beijing expansion would have taken place 900 BP whereas *modern* Beijing would have appeared only around 500 years ago [10]. Hirsch *et al*. suggested that east Asian and Philippines human populations carrying distinct MTBC lineages may have split only 240–1000 years ago [43].

The geographic origin of the emergence of L2 is as blurry as its dating. According to some authors, north-central and north-east China could have been the initial spreading centre [44]. According to others, based on differences in the prevalence of the *ancestral* L2 lineages in China and on the higher genetic diversity observed in the south-west province of Guizhou, south-China could be the craddle of L2 [8, 34]. Indeed, Guizhou counts 17 ethnical minorities and most of the acknowledged ethnic groups of China are located in this province. More generally, south-east Asia shows a higher human genetic diversity than the north-east Asia [45]. Arguing in favour of a south-China origin, the recent description of an ‘*asia ancestral 4*’ L2 sublineage, was done in the north of Thailand in Chiangrai, inhabitated since the 7th century and peopled by ethnic minorities originating from South-China [35].

Skeletal evidence of tuberculosis during the Bronze age was found in Korea and Japan [46, 47]. In Japan, one of the main characteristics of the tuberculosis history, especially in aged people that were not vaccinated by BCG, is the presence of still poorly characterised *ancestral* L2 strains [22, 26, 39, 42, 48]. MIRU-VNTR diversity had been shown earlier to be quite important in L2 isolates from Japan [44]. Other evidence based on MIRU-VNTR had suggested that some specific *ancestral* L2 strains could be endemic in Japan [49–52]. Since the publication of these studies, WGS of a few reference isolates were published in Kobe and Osaka however they were not mentioned in the simplified L2 phylogeny [28, 39].

We are currently developing the *TB-Annotator* project, a new computational genomics pipeline whose aim is to perform data-mining of MTBC genomic diversity using Sequence Read Archives (SRAs). We studied a set of L2 isolates from central Japan Honshu prefecture, Tochigi [42]. The 169 clinical isolates of *M. tuberculosis* we studied were from TB patients diagnosed in 2007 and in 2013 [42]. WGS data on these isolates was released after analysis using a bioinformatical pipeline, described as ‘CAST’ by Iwai *et al*. [53]. Since a comparative genomic analysis of these isolates with other *ancestral* L2 had not been performed, we included these genomes in our database. We also included SRAs labelled as *asia ancestral 4* [35]. Using our pipeline, we described the genetic characterisation of a new L2 sublineage from Japan, named *asia ancestral 5* (AAnc5), that appears to be exclusive from Japan for the time-being. We also assessed the characteristics of the AAnc4 sublineage described in Thailand and provide an updated global evolutionary framework of the L2 lineage [28].

## Material and methods

### Brief description of the *TB-annotator* pipeline

The full version of *TB-Annotator* is going to be released in another article (Senelle *et al*. in preparation). In brief, regarding the set-up of the platform, the processes are the following: SRAs of interest are selected and kept only provided a certain number of conditions that together reinforce the reliability of the data: they must have read length>75 bp, clean reads file must be at least 100 Mo, and CRISPR could be reconstructed using CRISPRbuilder-TB [54]. For each SRA, in addition to reconstructing the CRISPR-Cas region using CRISPRbuilder-TB and apart from collecting NCBI information on the genomes, two scripts successively perform the following tasks: (1) search for SNPs according to reference catalogues (Supplementary Table S1) totalling more than 50 000 SNPs, including drug-resistance related SNPs, phylogenetic SNPs as per 26 studies contributing to SNP-based classification [26, 28–30, 33, 55–75]; (2) search for additional SNPs in each isolate based on H37Rv reference sequence; (3) look for the presence/absence of H37Rv genes as annotated in mycobrowser (https://mycobrowser.epfl.ch/), (4) look for the presence/absence of deletion regions; (5) identify insertion sites of all known insertion sequences in MTBC. The CRISPR locus is rebuilt semi-automatically using a dedicated and previously published script (43/68/360 Spacers format) with an assignment of a Spoligo-International-Type (SIT) tag; the application produces an ordered list of spacers/repeat with variants and IS*6110* insertion sequences if present [54, 76, 77].

### Selected genomes

We downloaded a set of 680 SRAs; these samples were selected to represent TB genomic diversity (L1 to L9) described so far, including recent landmark papers [29, 30]. The list of these SRAs is shown in Supplementary Table S2 **(**list of 680 SRA including 190 L2 SRA); the database was built to represent all L2 sublineages except for the Pacific RD150 sublineage. From [35], we selected 28 SRAs labelled AAnc4. From [42], we initially included 158 SRAs, however 57 SRAs for which the coverage was either too weak or for which it was impossible to rebuild the spoligotype using CRISPR-builder-TB were discarded [54, 77].

### Bioinformatics and phylogenetical methods

Final scripts allow to produce a phylogeny based on the list of studied characters and using RAxML and SplitsTree [78, 79]. All computations were performed on the ‘*Mésocentre de Franche-Comté*’ supercomputer facilities (141 nodes, 2292 cores, 9,27 To memory, 74,2 CPU Power TFlops, 66,4 GPU TFlops), using adequate commands. Apart from the results shown in the Supplementary Table S3, a final phylogenetic tree is displayed graphically and proprietary python scripts allow interactive queries to be made and results to be shown [79]. The current *TB-Annotator* version includes 6009 genomes and confirmed our results (Senelle *et al*. in preparation).

### SNP-based classification of L2 sublineages

**Fig. 1.**
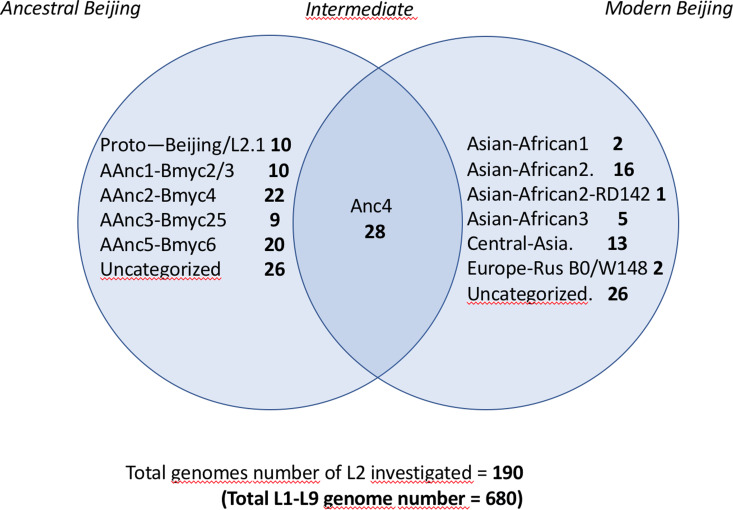
Venn diagram showing the classification of the 190 L2 SRA studied; note the linking or ‘intermediate’ status of AAnc4 between ancestral and modern strains.

In order to assign the 680 selected SRAs into known lineages and sublineages, we used the reference list of markers defined in Supplementary Table S1. A simplified Venn diagram shows the classification of the 190 L2 SRAs into 125 ancestral and 65 modern isolates (Fig. 1). SNP-based classification results on the 190 SRA of the L2 isolates, as produced by *TB-Annotator* are found in the Supplementary Table S3.

### Methods and dataset used to define a new *Asia ancestral 5* sublineage (AAnc5)

**Fig. 2.**
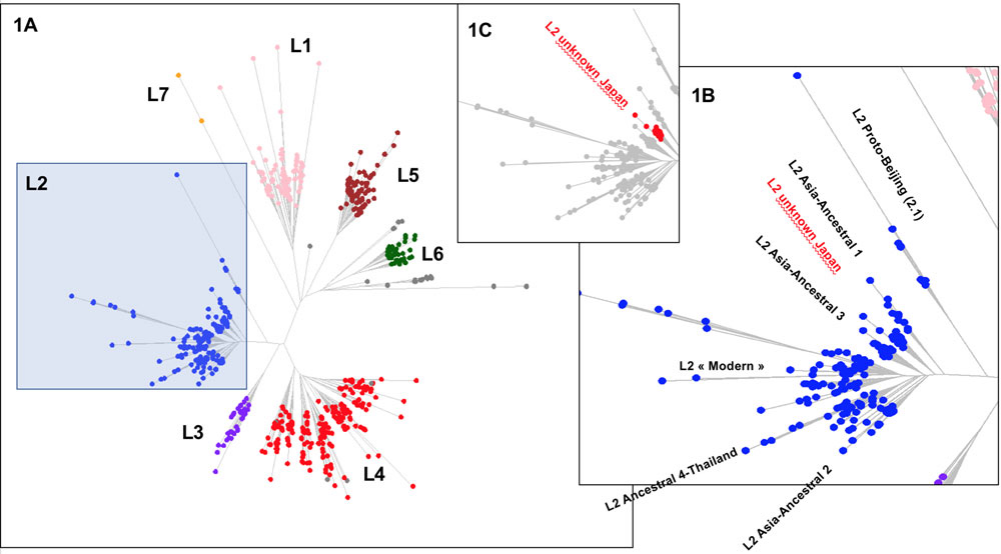
Left part (a); *TB-Annotator* unrooted phylogenetic tree on 680 SRA-derived data. L2 samples are shown in blue. Right part (b); close-up on the Lineage 2 with all known branches named except in red the new unknown *ancestral* Japan sublineage we designated as *asia ancestral 5*. Central part (c); focus on the unknown Japan lineage.

After the assessment of existing known MTBC sublineages (Fig. 2a and b), the unknown branch (Fig. 2c) was investigated in detail: *in Silico* CRISPR locus reconstruction using CRISPR-builder TB [54, 77], *in Silico* MIRU-VNTR using CAST [53]; SNPs assessment and intra-cluster distance with *TB-Annotator*; AAnc5 was shown to originate only from SRAs from Japan isolates (Bioproject PRJDB3875). The bioinformatical pipeline, based on its graphical user interface, allows to select and display new exclusive or shared SNPs and specific genetics characteristics, that were further investigated. Previous genomic markers extracted from Wada *et al*. 2012 were compared to our results and are found in Supplementary Table S4 [39].

## Results

### Discovery of a new L2 sublineage

We implemented a representative set of SRAs of L2 including 101 extracted from the 169 samples study from the Tochigi province study, onto the *TB-Annotator* platform [42]. The classification of the 190 L2 studied genomes is shown in a simplified Venn diagram (Fig. 1**)** and the global phylogenetical tree produced is shown on Figure 2a–c. All samples carried the L2 defining SNP (G497491A) and were found in the same phylogenetic branch (Fig. 2a). Based on SNP search, and as shown on Figure 2b and c and in more detail in Supplementary Table S3, among the *ancestral* Beijing (*n* = 125) we found Proto-Beijing (L2.1; *n* = 10), *asia ancestral 1* (L2.2.2-AAnc1, *n* = 10), *asia-ancestral 2* (AAnc2, *n* = 22), *asia ancestral 3* (AAnc3, *n* = 9), *asia ancestral 4* (AAnc4, *n* = 28), and an unknown branch (suggested as *asia ancestral 5* or AAnc5, *n* = 20); there remained 26 unclassified *ancestral* Beijing. Figure 1 and Supplementary Table S3.

Ninety-three isolates (including AAnc4), were part of L2.2.1 and were all harbouring a *mutT2* G1286766C that is traditionally defining a ‘*modern Beijing*’ characteristic SNP. Out of these 93, however 65 samples only (excluding AAnc4) were harbouring the second *modern* L2 signature, i.e. the C1477596 T SNP in *ogt*, while the 28 AAnc4 did not harbour this SNP [28, 35]. Hence, the designation of *modern* L2 should be kept for isolates harbouring these two SNPs simultaneously and not only the *mutT2* G1286766C polymorphism.

**Fig. 3.**
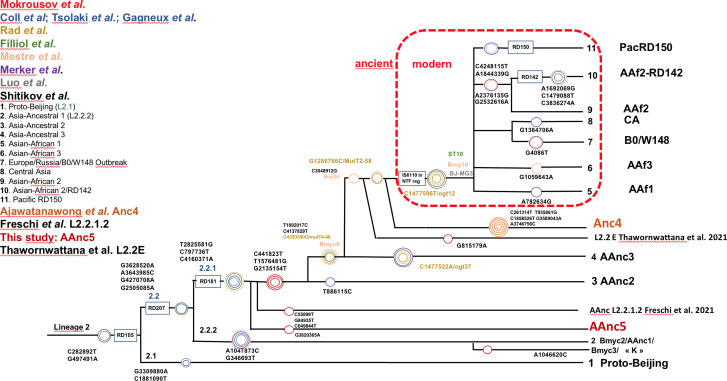
Unified Mtb lineage 2 dendrogram representing the current L2 sublineages with some of their defining SNPs or genetic markers. The colour code tries to superimpose with each author, the size of the circles is arbitrary; this tree tries to provide a simplified unified evolutionary scheme however does not claim to be representative of the full diversity of L2 (redrawn and improved from Shitikov *et al*., 2017).

The other *modern* Beijing (*n* = 65), are further split into *asian african 1* (*n* = 2), *asian african 3* (*n* = 5), *asian african 2* (AAfr2, *n* = 16), *asian african 2*-RD142 (AAfr2-RD142, *n* = 1), *central asian* (*n* = 13), Europe/Russia B0/W148 outbreak (*n* = 2) and there were a remaining 26 *unclassified modern Beijing* isolates, that did not fit to any described modern sublineage definition, and that were not investigated further in this study. No Pacific RD150 isolates was included in this study. We present an improved unified L2 classification scheme that includes the recent discovery of AAnc4, our own AAnc5, as well as the ‘L2.2.1.2’ [30], the ‘K’ strain (a member of AAnc1) [80], and the L2.2.E [81], as shown on Figure 3.

The reanalysis of the phylogenetical SNP set described by Shitikov *et al*. confirmed all the phylogenetical SNPs specific of the *ancestral* L2 sublineages up to the definition of the *modern* L2. The *mutT2* SNP (G1286766C) is a good phylogenetic marker as it is present only in L2 modern sublineages whereas T1892017C, C4137829T and C4393590G (*mutT4*) are present in some *ancestral* sublineages. It is clear from the SNPs results and from the phylogenetical tree shown in Figure 2 that the AAnc4 branch could be called intermediate, as suggested by the team who discovered it, and is neither a *bona fide* modern L2 sublineage nor a truly *ancestral* one [35]. Conversely, the 20 isolates that we studied from Japan, further designated as AAnc5, all Fulfill the *ancestral* L2 criteria since they do not possess the expected *mutT2* (58) SNP nor any other characteristic of modern Beijing. They also branch before AAnc4. In all cases, the clear-cut split of L2 isolates that either possess none, one, or the two *mutT2* (58) and *ogt* (12) SNPs confirms that these two markers are excellent phylogenetical ones. Based on the results obtained, we thus adapted the unified classification scheme of Shitikov *et al*. to include some of the new recently described sublineages (Fig. 3**)**. Among those, are the ‘K’ strain (part of AAnc1), the ‘L2.2.1.2’, the L2.2.E [30, 31, 80], and the AAnc5 recently designated as L2.2.A [31].

### Description of a new *Asia-ancestral 5* (AAnc5) Japan sublineage

#### Snp-based Results

We further characterized the Japan *asia ancestral 5* sublineage (AAnc5) (*n* = 20) that branches between AAnc1 and AAnc3 (Fig. 2b and c). These isolates can all be defined by many exclusively shared SNPs. The SNP-based classification produced by the *TB-Annotator* pipeline on the 190 L2 genomes can be found in Supplementary Table S3. In the future versions of *TB-Annotator*, reports will be downloadable from a dedicated website (Senelle *et al*. in preparation). The list of 42 exclusive SNPs, found in 16 of these 20 isolates is shown in Supplementary Table S4. An interesting observation is that all the AAnc5 strains harbour a non-synonymous mutation at position 765140 (G->C) in *rpoC*. Three other genomes (2 in other L2 sublineages and one in a L4 sublineage) also carry this *rpoC* mutation suggesting independent acquisition.

A pairwise distance matrix between AAnc5 isolates was also computed (Supplementary Table S5**)**; the current pipeline systematically computes the intra-branch SNP distance for clusters or selections of interest below 100 SRAs (results not shown). The pairwise distance between the AAnc5 samples shows a minimum of 166 SNPs (between DRR157280 and DRR157281) and a maximum of 439 SNPs (between DRR130203 and DRR034366) inside AAnc5 isolates, thus excluding recent transmission (Supplementary Table S5). Assuming a mean 0.3 SNP mutation rate per year per genome, these strains might have diverged approximately 250 to 600 years ago from their MRCA. If we accept the suggestion of timing of AAnc4 emergence or expansion around the 7th century in Thailand, (start of Chiangrai), then AAnc5 could have been introduced into Japan earlier, in line with archaeological information [35, 46, 47]. A second list of 46 exclusively shared SNPs, shared between the two most distant isolates on a specific subbranch of AAnc5 (DRR034381 and DRR130203, pairwise distance: 417 SNP) is also shown in Supplementary Table S5.

#### *In silico* spoligotyping and reconstruction of the CRISPR locus structure of AAnc 5 using *TB-annotator*, IS*6110* copy number and insertion sites

The twenty studied AAnc5 isolates showed 6 different spoligotype patterns as reconstructed by CRISPR-builder, which was an unexpected result for an L2 sublineage (Supplementary Table S6); most of these patterns have been previously described in the SITVIT^4^ database (SIT1, SIT190, SIT269, SIT1364, SIT1674), however one remained undefined as SIT”X”. No SNP variants were found in spacers and repeats, but three isolates exhibited duplications: a duplication of sp65 for DRR034478 and SRR130160, and of sp50 for DRR034476 (Supplementary Table S6). The phylogeny that can be derived from the reconstruction of the CRISPR-Cas structure confirmed the SNPs results: it reveals sporadic deletions of *cas* genes, Rv2807c, Rv2808c and Rv2813c in some isolates (Supplementary Table S7).

AAnc5 strains were harbouring from 14 to 22 IS*6110* copies, and two specific copies were found in almost all these L2 isolates and not in other L2 sublineages: one copy was found at position 1724419 in Rv1527c (found in 15 of these isolates) and the second one was found at position 2041756 (found in 19 of these isolates) (Supplementary Table S6). DRR034455, DRR034471 and DRR034476 were showing the same CRISPR structure, however harboured different missing genes (see next paragraph). Using *TB-Annotator*, 14 of the AAnc5 isolates were predicted to be drug-sensitive and four were harbouring mono-resistant mutations, two were MDRs (Supplementary Table S8).

#### Missing genes

Six TB isolates among the 20 AAnc5, apart from showing classical deletions (RD105, RD207, RD181 and PhiRv1), were harbouring specific missing genes: as an example, DRR034363 had Rv1081 to 1084c deleted, DRR034416 was missing Rv1523 to Rv1526c. (Supplementary Table S7). These deletions confirm that phylogenetically linked MTB genomes sometimes harbour strain-specific dependent deletions due to recombination events.

#### *In silico* VNTR copy number computation using CAST and comparison with other isolates from previous studies

No specific 15 + 9 VNTR signature could be obtained from *in Silico* VNTR typing using CAST for any of the AAnc5 isolates [52, 82]. ETRC, QuB26 and QuB4156 could never be *in Silico* predicted. Depending on SRA quality, between 6 and 20 VNTR could be predicted (Supplementary Table S9). The VNTR results showed slight variation between isolates; eleven VNTR Loci were invariant in this collection (MIRU04, MIRU10, MIRU16, MIRU20, Mtub29, Mtub30, ETRB, MIRU24, MIRU27, Mtub34, MIRU39) whereas nine loci showed variation (MIRU02, MIRU40, Mtub21, QuB11b, ETRA, MIRU23, MIRU26, MIRU31, Mtub39). When comparing with an in-depth VNTR study performed earlier, AAnc5 was shown to belong to M10 or M37 respectively found in Russia and Singapore [44]. When comparing with a set of 5 reference Japanese isolates (A05N056, ID381, 4558, 4994, 4991/M) that were described to represent the main L2 sublineages found in Japan, ID381 was sharing the same VNTR copy number with AAnc5 on 9 loci (Supplementary Table S9) [22, 39]. When comparing *in Silico* VNTR results with previous VNTR results from published studies in Korea, on the ‘K’ strain, known to belong to AAnc1, we retrieved relatively poor similarity [38, 80] (Supplementary Table S9).

#### Comparison between *TB-annotator* and CAST server results on prospective drug susceptibility testing and on spoligotyping results

When comparing the drug-susceptibility testing results obtained using either the *TB-Annotator* pipeline or CAST, they were identical (Supplementary Tables S8 and Table S9). Identical results were also obtained on the classical 43 spacers-format spoligotype reconstruction with a minor and yet unexplained discrepancy on a single spacer of a single isolate, DRR034366, for which CAST predicted SIT250 whereas *TB-Annotator* predicted SIT290 (Supplementary Table S9).

#### AAnc5 is identical to the G3 endemic L2 ancestral strains cluster in Japan

We compared the 42 SNPs table found in the AAnc5 sublineage (Supplementary Table S4) with the ones found in sequences of 5 Japanese reference isolates, compared to the K1-K2 epidemic strain [39]. According to the definition made by these authors 5 L2 subgroups (G1/2, G3, G4, G5/6 and M) could be defined in Japan based on 10 phylogenetically selected SNPs. From the ID381 strain, a member of the G3 genetic group, described for the first time in Kobe and Osaka in 2006 [39], looking at G3 specific SNPs set (Supplementary file of [39]), and comparing it with AAnc5 SNPs set, we concluded that the G3 group was new and did not fit with the former Shitikov *et al*. classification scheme. Indeed, AAnc1, AAnc2 and AAnc3 were found to respectively match with G1/2, G4 and G5/6 in [39], however no equivalent was found for G3.

By comparing SNPs lists, we found that the Tochigi province AAnc5 cluster of strains was sharing 36 out of 42 SNPs with the G3:ID381 reference strain found in Kobe and Osaka (Supplementary Table S4). 6 SNPs only (C587945T, G765140C, G1202113A, AGGGAG1476812A, G3148446C and G3820365A) were not found in the ID381 strain. We concluded that the Tochigi strains were highly likely to be historically related to the Kobe and Osaka G3 group described in 2006 through the reference isolate ID381. Accordingly, we propose to retain the common SNPs described by Wada *et al*. and this study as characteristic of the AAnc5 to fit with Shitikov *et al*. nomenclature. We positioned both AAnc5 and AAnc4 in the global schematic L2 tree [28, 35]. By digging more in-depth into the comparative SNPs list between our study and the former Kobe-Osaka study, we found that DRR034489 was the closest isolate to the G3 ID381 reference sharing 40 more SNP exclusive to the G3 group, whereas another cluster of 3 genomes were more distant but were sharing 15 more SNPs with ID381 (results not shown). As mentioned above, two very distant genomes, DRR034381 and DRR130203 (417 SNPs pairwise distance) where also sharing 46 additional SNPs that were not found elsewhere (Supplementary Table S5).

## Discussion

We describe in this paper a historical endemic *ancestral* sublineage of L2 based on samples collected in central Japan, Tochigi Prefecture, former Shimotsuke province, that we named AAnc5. This sublineage is highly related to the Japanese G3 group defined in 2012 [39] and assumed to be named L2.2.A in a recent review [31]. Our results strengthens the phylogenetical relevance of this sublineage into the global L2 evolutionary history and shows that it was transmitted historically in several Japanese cities. The chronology of the emergence of this sublineage relatively to other L2 sublineages was positioned in Shitikov's L2 diversification scheme. Its position relative to previously described lineages clearly showed that it should be qualified as *ancestral* according to the current definition of this terminology and diverged from other Beijing sublineages shortly after AAnc1.

Tuberculosis is very ancient in mankind history, however it is still impossible to definitively date its emergence in Asia [10]. Early TB outbreak history in Japan could be related to migrations of people from the 5th century BC to the 3rd century AD [46, 47]. Tuberculosis was known to be present in ancient time in Japan under the name of *rôga*, that was used in Chinese medicine [81]. A link between this disease and TB, as known in the western medicine, was described in 1857 by a medical doctor, Ôgata Kôan, 3 years before the opening of Japan [81]. There are many traces in Chinese medical history texts of a disease that can be identified as tuberculosis [83]. The historian Fan Xingzhun gave some clues in his brief study on the history of this disease in China [84]. His analysis of Chinese sources leads him to point out that many terms associated with symptoms suggestive of tuberculosis, appear in ancient sources. The *Classic of Mountains and Seas* (*Shanhai Jing* 山海經, 4th–3th century BC) describes a remedy to cure the ‘*luo* 瘰’, i.e. the scrofulae. However Fan Xingzhun admits that these symptoms may not be specific. He notes that other terms suggestive of the disease appear in other ancient sources such as the *Classic of Poetry* (*Shijing* 詩經 gathering texts spanning from the 11th to the 5th century BC) or in the *Mengzi* (孟子 4th century BC), that Su You 蘇游, a Tang Dynasty (618–907) author, holds as synonyms. Fan Xingzhun stresses that these terms (*zhai* 瘵，*mo* 瘼，*chuanshi* 傳尸 (literally: corpse that transmits), *shoubing* 瘦病 (literally: thinness disease), *zhuanzhu* 轉注，*fulian* 伏練, *guzheng* 骨蒸 (literally: bones filled with hot steam or hot bones) are often associated with a description of states of extreme fatigue and emaciation in early dictionaries and medical books. The ‘*zhai* 瘵’ ideogram, in particular, that has become one of the most popular to describe, between others, tuberculosis, is defined in the earliest dictionaries as a disease whose main characteristic is weakness, and, later, as a disease that leads the patient ‘to toss and turn restlessly’ until ‘dying of weakness’. While Fan Xingzhun agrees that all these terms may not necessarily correspond to tuberculosis, the historian nevertheless finds it very likely that the evidence he found in a 14th century book describes a case of pulmonary tuberculosis: ‘*Under the Song* (d.960-1127), *Shi Dezun, when he was 50 years old got the exhaustion and weight loss disease, he was tossing and turning restlessly until he became very emaciated’* [84]. Further, Fan Xingzhun hypothesise (p.97) that tuberculosis prevalence was low in ancient times. However in the *Ancient Book of Tang (Jiu Tangshu*, 舊唐書 941 AD) it is reported that: « *under the Wude reign (618–626), in Guanzhong (Shaanxi province), many have the ‘hot bone disease’* 骨蒸 », a testimony that argues in favour of a tuberculosis epidemic. As the term ‘hot bone disease’ (骨蒸) is already mentioned in the medical Canon ‘The Yellow Emperor's Canon of Medicine’ (*Huangdi Neijing* 黃帝内經, 2BC-2AC), Fan Xingzhun wonders about the possibilities that tuberculosis could have already been epidemic at that time [84].

Going *in-depth* into the complex historical phylodynamics history of all MTBC lineages was made possible using the *TB-Annotator* pipeline that analyses more than 50 000 characters including repeated sequences and SNPs [29, 30, 85–88]. With an increasing publicly available number of SRAs, it becomes feasible to disentangle all the threads between an ancient and recent historical event that shaped today's TB pandemia, and to understand its relation to ancient/modern population migrations [10, 89, 90].

Among the set of AAnc5 characteristics, we describe a non-synonymous mutation in *rpoC*. It is well known that *rpoC* mutations are compensatory mutations mainly found in epidemiologically successful isolates that also contain specific *rpoB* mutations [12, 15, 91]. The *rpoC* mutation could be an adaptative trait that explains the epidemiological success of MDR-TB L2 isolates in outbreaks such as the Central Asian or the B0/W148 outbreak in Russia [6, 92] and the epidemiological success in Georgian prisoners [15]. In the central Asian L2 clade, *rpoC* mutations may arise within the entire gene and these SNPs are found in epistasia with *rpoB* mutations [6, 12, 91]. In our study, the *rpoC* mutation, except for the six most recent last isolates, was found in drug-susceptible isolates; hence it is difficult to consider such a mutation as adaptative or compensatory, but rather as a phylogenetical marker of AAnc5 [15]. Since this SNP was not described in the G3 group from Kobe and Osaka [39], it could be interesting in the future to try to search if there is a significant statistical difference between drug-resistance emergence in one or the other cluster group of isolates. A recent paper in prisoners in Georgia showed that compensatory mutations and patient incarceration were two independent factors associated with the increased transmission, that create a ‘*perfect storm*’ for MDR-TB transmission [15]. The precise physiological or fitness consequences of this non-synonymous *rpoC* mutation in drug-sensitive isolates was not investigated in this study but it could have also functional consequences [12]. L2 evolution includes the early evolutionary history of AAnc5 and could be linked to yet unknown mutator effects specific of some L2 sublineages [11].

Dating of strains diversification may also give some clues on the social and demographic conditions that fostered past epidemics. Calibration of the molecular clock is difficult according to samples, time-frames and lineages (between 0.04 and 2.2 SNPs per-genome-per-year) [93]. Dating can be comforted if an adequate correlation exists between historical, genomic, epidemiological and demographic facts [90]. In a former similar study, we tested three scenarios to date the MRCA of an L4.2 sublineage occurring in Japan and Turkey, and confronted historical, anthropological, human genetics, paleopathological and genomics results [68]. Here, according to the consensus molecular clock of TB on the middle term, the time frame of coalescence of AAnc5 sublineage landmarks could be between 280 and 310 years ago before present for the closest isolates, and 760–800 years ago for the farthest ones. We based this estimate on L4 and not on L2 mutation rates and hence, are very cautious.

The Tochigi province is famous for its great copper Ashio mine, whose exploitation started beginning of the 17th century. One of our estimations of the expansion date of the AAnc 5 is compatible with the opening of the mine in Ashio [94]. Ashio mine could have been one location of AAnc5 expansion and diversification. Looking at the historical spreading dynamics of the AAnc5 group in Japan could be done in the future with the help of Japanese investigators by looking at finer gradients of all prevalent L2 *ancestral* sublineages found in Japan.

Another more recent origin of Japanese AAnc5 could be the importation of *ancestral* L2 clones by forced workers from Korea or China who were made to work in the mining industry from 1939 till 1945 [81]. Indeed 300 000 Koreans and 38 935 Chinese workers, mostly men were forced to work for Japan during these years [81]. However given the high SNP number accumulated inside AAnc5, this hypothesis seems unlikely. One limitation of this study is that we could not investigate the potential distribution of G3/AAnc5 isolates within all Japan, however our formal proof that G3 and AAnc5 are linked and deeply rooted is the first insight into a complex history. Future study could try to dissecate further, using a combination on recent statistics on the total of epidemic cases, in combination to genomic characterisation and geographical distribution, the global and the local history of L2 *ancestral* lineages in Japan [95].

The existence of a relatively high diversity of isolates specific to Tochigi and Kobe and Osaka, reminds of historical transmission restricted to a circumscribed area. Of course, such a picture of an endemic past outbreak is more easily observed in islands settings as was shown recently in New Zealand in Maori people who are hosting a specific ‘CS’ (Colonial S-type) L4.4 Sublineage [90]. Islands are excellent settings to distinguish endemic from imported species and tuberculosis history is definitively linked to human migration and to local and global demographic history [96]. According to the most recent review on L2 population structure based on more than 5000 genomes, AAnc5 or L2.2.A *‘is the most basal clade of L2.2.1 and comprises isolates almost entirely from Japan. The deep- branching structure of L2.2.A is suggestive of a previously unrecognized endemic strain*’ [31]. Since Japan is an island, and a country with a very low current tuberculosis prevalence rate (13 per 100 000 in 2017), it is an excellent setting to identify the historical events linked to past tuberculosis outbreaks [68]. The increasing number of available genomes allows more and more L2 sublineages to be discovered, however their intimate historical and epidemiological relationships remains to be studied in more detail [30, 31].

## Conclusions

Thanks to *TB-Annotator*, a new bioinformatical pipeline that analyses large amounts of the information contained in SRAs, we mapped an endemic L2 *ancestral* sublineage from Japan onto the global MTBC phylogeny, and we designated it as *asia ancestral 5* (AAnc5), and showed that it was linked to the formerly described G3 group in Kobe and Osaka, and now designated as L2.2.A. This sublineage now appears together with some among the most recent one's in a unified evolutionary scheme. AAnc5 possesses many specific characters allowing it to be distinct from all other *ancestral* sublineages described so far in L2. This finding opens new ways of research, to look for the history of L2 in south-east Asia.

## Data Availability

All data are publicly available either as SRA Accession numbers on the NCBI or EBI website, or provided herein. The source code of the *TB-Annotator* pipeline is will be published elsewhere (Senelle *et al*. in preparation).

## Footnotes

1 Restriction Fragment Length Polymorphism

2 Mycobacterial Interespersed Repetitive Units-Variable Number of Tandem Repeats

3 Spoligotyping-international-Type

4 Spoligo-International-Database (http://www.pasteur-guadeloupe.fr:8081/SITVIT2/)

## Supplementary material

For supplementary material accompanying this paper visit http://doi.org/10.1017/S0950268822000048. click here to view supplementary material
